# Whole exome sequencing identified a homozygous novel variant in *DOP1A* gene in the Pakistan family with neurodevelopmental disabilities: case report and literature review

**DOI:** 10.3389/fgene.2024.1351710

**Published:** 2024-05-16

**Authors:** Wei Zhang, Muhammad Tariq, Bhaskar Roy, Juan Shen, Ayaz Khan, Naveed Altaf Malik, Sijie He, Shahid Mahmood Baig, Xiaodong Fang, Jianguo Zhang

**Affiliations:** ^1^ College of Life Sciences, University of Chinese Academy of Sciences, Beijing, China; ^2^ BGI Genomics, Shenzhen, China; ^3^ National Institute for Biotechnology and Genetic Engineering College, Pakistan Institute of Engineering and Applied Sciences (NIBGE-C PIEAS), Faisalabad, Pakistan; ^4^ Hangzhou Institute of Medicine (HIM), Chinese Academy of Sciences, Hangzhou, Zhejiang, China; ^5^ China National GeneBank, BGI-Shenzhen, Shenzhen, China; ^6^ Hebei Industrial Technology Research Institute of Genomics in Maternal and Child Health, Shijiazhuang, China; ^7^ Clin Lab, BGI Genomics, Shijiazhuang, China

**Keywords:** neurodevelopmental disorders (NDDs), myelination, DOP1A, Mon2, proteolipid protein (PLP), myelin-associate glycoprotein (MAG)

## Abstract

**Background:**

Hereditary neurodevelopmental disorders (NDDs) are prevalent in poorly prognostic pediatric diseases, but the pathogenesis of NDDs is still unclear. Irregular myelination could be one of the possible causes of NDDs.

**Case presentation:**

Here, whole exome sequencing was carried out for a consanguineous Pakistani family with NDDs to identify disease-associated variants. The co-segregation of candidate variants in the family was validated using Sanger sequencing. The potential impact of the gene on NDDs has been supported by conservation analysis, protein prediction, and expression analysis. A novel homozygous variant *DOP1A(NM_001385863.1):c.2561A>G* was identified. It was concluded that the missense variant might affect the protein-protein binding sites of the critical MEC interaction region of DOP1A, and DOP1A-MON2 may cause stability deficits in Golgi-endosome protein traffic. Proteolipid protein (PLP) and myelin-associate glycoprotein (MAG) could be targets of the DOP1A-MON2 Golgi-endosome traffic complex, especially during the fetal stage and the early developmental stages. This further supports the perspective that disorganized myelinogenesis due to congenital *DOP1A* deficiency might cause neurodevelopmental disorders (NDDs).

**Conclusion:**

Our case study revealed the potential pathway of myelinogenesis-relevant NDDs and identified *DOP1A* as a potential NDDs-relevant gene in humans.

## Introduction

*DOP1A* is a protein-coding gene involved in the DOPEY family with a leucine zipper-like domain. *DOP1A* is located on chromosome 6,NC_000006.12 (83067671.83171350) in humans. The homologous gene *Dop1A* was first identified in the filamentous fungus *Aspergillus nodulins* with a leucine zipper-like domain at the N-terminal and two adjacent leucine zipper-like domains at the conserved region in the C-terminal, which was considered critical for the correct cell morphology and spatiotemporal organization of multicellular structures (Pascon and Miller, 2000). There are two Dopey orthologs in mammals, *DOP1A* and *DOP1B*. It has been reported that *DOP1A* triggers body tremors, absence-like seizures, and abnormal myelination in null mutant rats (Tanaka et al., 2012; 2014), but no human studies have been carried out. Lei Lu et al. reported that DOP1A and MON2 assemble into a complex and localize in Golgi, endolysosomes, and endoplasmic reticulum to facilitate membrane transport (Mahajan et al., 2019; Zhao et al., 2020). The *DOP1B* gene has been considered a candidate gene for human brain abnormalities and mental retardation in Down syndrome and Peters anomaly (Rachidi et al., 2006; Darbari et al., 2020). DOP1B has also been reported to assemble with MON2 and affect SNX3-retromer-mediated Wntless sorting (McGough et al., 2018).

Neurodevelopmental disorders (NDDs) are disabilities caused by dysfunction of the peripheral and central nervous systems (Mullin et al., 2013). Neurodevelopmental disorders are a large class of diseases with clinical heterogeneity, characterized by attention-deficit/hyperactivity problems, learning disabilities, intellectual disability, cerebral palsy, dyskinesia, seizures, impairments in vision/hearing, autism spectrum disorder (ASD), communication disorders (Thapar et al., 2017), and other developmental delays, despite their mental problems. Neurodevelopmental disability symptoms probably vary or evolve in the process of children’s growth. Although the etiology of many developmental disorders remains unclear, poor maternal immune activation is a high-risk factor for this disease (Hall et al., 2023). Genetics plays a major role in it, and those disabilities caused by genetics are always severe and permanent (Morris-Rosendahl and Crocq, 2020). Intellectual disability (ID) is one of the most prevalent symptoms of hereditary developmental disabilities (Yang et al., 2022). Studies have shown that abnormal neurons are associated with the pathogenesis of neurodevelopmental disorders. Neuron axons and dendrites with highly polarized cellular morphology are functioning as neuron circuits, in which dendrites are highly branching with a distinguished pattern associated with different neuron cell types (Lefebvre et al., 2015). Abnormalities in dendrites are strongly associated with both neurodegenerative disorders and intellectual disabilities (IDs) (Emoto, 2011). Dendritic spines are specialized structures found exclusively in neurons that serve the crucial function of receiving and integrating input from excitatory synapses. These structures undergo extensive remodeling during the developmental and pathological stages.

Perturbations in the essential processes responsible for dendritic elongation and formation can lead to aberrant dendritic morphogenesis and dysfunction. These aberrant processes are frequently observed in diseases associated with intellectual disabilities (De Rubeis et al., 2012; Kulkarni and Firestein, 2012; Torres et al., 2018). Besides the neuron fiber, the myelin sheath has been widely recognized as crucial for nerve conduction and other neuronal functions. Within the central nervous system (CNS), oligodendrocytes (OLGs) are responsible for producing myelin sheaths that ensheath axons. The myelination of neurons enables high-speed conduction from axons to dendrites and enhances the functional efficiency and complexity of the nervous system (Hartline and Colman, 2007). According to Zalc et al., it has resulted in a smaller but more complex nervous system in vertebrates (Zalc et al., 2008). In humans, the white matter of the brain is primarily composed of myelinated nerve fibers, which comprise over 50% of the central nervous system.

In the central nervous system, proteolipid protein (PLP) is the predominant component of the myelin sheath, accounting for 50% of the total myelin protein. The myelin basic protein (MBP) makes up 30% of the myelin protein, whereas the myelin-associated glycoprotein (MAG) accounts for 10% (Boggs et al., 2006). Therefore, any changes in the levels or structure of these critical proteins can result in myelin dysfunction and contribute to neurodevelopmental disorders.

The pathogenesis of NDDs is still unclear, but many studies have revealed that genetics plays an important role. The causes of NDDs are numerous including non-genetic and genetic factors. Environmental factors such as prenatal and postnatal infections, perinatal hypoxia, maternal diseases like diabetes or phenylketonuria, prematurity, and environmental factors (such as iodine deficiency and malnutrition), metabolic causes, and teratogenic factors (such as exposure to alcohol, drugs, environmental chemicals) are often related to the mother’s lifestyle and the level of medical care.

The genetic factors of NDDs also exhibit high heterogeneity, including large chromosomal abnormalities, chromosomal microdeletions and duplications, and monogenic diseases. Monogenic diseases can be further categorized based on their mode of inheritance into X-linked, autosomal dominant, and autosomal recessive dominant (AR). Over thousands of genes have been identified to be connected with NDDs (Kochinke et al., 2016).

In this study, whole-exome sequencing (WES) was performed in a consanguineous Pakistani family with NDDs, and a novel homozygous variant DOP1A *(NM_001385863.1)* :c.2561A>G was identified in the *DOP1A* gene. This novel homozygous variant might cause instability in the possible Golgi-endosomes protein traffic DOP1A-MON2 and result in disorganized myelinogenesis in nerve fibers, which ultimately manifests as NDDs in this affected family.

## Materials and methods

### Subjects

The National Institute for Biotechnology and Genetic Engineering collected a sample of families who suffer from intellectual disabilities in Pakistan. The probands’ biological parents were consanguineous. Among the probands and their affected relatives, deficiencies in movement, linguistic competence, and adaptive behavior were commonly observed (Table 1). In addition, some patients exhibited co-occurring symptoms such as epilepsy or skeletal abnormalities. Three peripheral blood samples were drawn from participants who, or whose guardians, provided consent. Informed consent for the study was obtained, and sample collection and information records were authorized. All procedures were authorized by informed consent from study participants. The study was conducted according to the principles outlined in the Declaration of Helsinki, and all protocols, consent forms, and procedures for the collection of samples were approved by the local institutional review board, specifically the Research Ethics Committee of the National Institute for Biotechnology and Genetic Engineering in Faisalabad, Pakistan, on 20 June 2016.

**TABLE 1:** The genotypes and disease status of relatives.

Sample ID	Relationship	Genotype (DOP1A:c.2561)	Status
Ⅲ-1	The proband’s mother	GA	Unaffected
Ⅲ-2	The proband’s father	GA	Unaffected
Ⅲ-3	The proband’s aunt	AA	Unaffected
Ⅲ-4	The proband’s uncle	AA	Unaffected
Ⅳ-1	The proband	GG	Affected
Ⅳ-2	The younger brother of the proband	GG	Affected
Ⅳ-3	The proband’s unaffected sibling	GA	Unaffected
Ⅳ-6	The proband’s unaffected cousin	AA	Unaffected

### Whole exome sequencing

WES was performed for two probands and their father. Peripheral blood white cells were used to extract whole genome DNA. The exomes were enriched and indexed using BGI exon 59M V4 kits. Sequencing was performed as 100 bp paired-end reads on BGISEQ-500 systems, which used combinatorial Probe-Anchor Synthesis and improved DNA Nanoballs technology (Fang et al., 2018). The data from this study have been deposited in the CNGB Nucleotide Sequence Archive and are publicly available (CNSA: https://db.cngb.org/cnsa;accession number CNP0002171).

Adapter sequences were removed, and low-quality reads, defined as those containing excessive Ns or low base quality, were filtered out. The resulting high-quality “clean reads” were aligned to the human reference genome hg19 using both BWA and SOAP alignment tools. Variation detection was processed using the pipeline from the Genome Analysis Toolkit.

All variants were then annotated for transcripts and mutation types. Quality control was an integral part of our pipeline, ensuring that only high-quality data was used throughout the analysis process, including read alignment and variant calling. The SNPs were called by GATK HaplotypeCaller using a joint calling strategy, which combines information from multiple samples to improve accuracy.

Just as the filtering parameters commonly used (Han et al., 2020; Zhang et al., 2020), we use the following parameters to filter variants here: 1) The VAF should be limited to less than 0.005 and must be segregated by heredity. 2) The variants should be located in protein-coding regions. 3) The variants that are classified as benign or likely benign according to the ACMG guidelines (Richards et al., 2015; Oza et al., 2018) should be eliminated, but those of uncertain significance and above could be candidates. 4) The loci should be conserved across mammals by multiple sequence alignments. 5) The variants should be co-segregated with other family members according to the Sanger validation. Twelve variants were conserved across species, and Sanger was used to validate them. Finally, the variant *DOP1A(NM_001385863.1):c.2561A>G* was co-segregated within the family by Sanger validation (Table 1; Supplementary Figure S1). Both parents were heterozygous for the *DOP1A(NM_001385863.1)*:*c.2561A>G*, and the proband inherited the variant from both parents, resulting in a homozygote. The presence of the same rare variant in both parents might suggest consanguinity.

### ACMG classification

The variants were classified according to the American College of Medical Genetics and Genomics (ACMG) guidelines (Richards et al., 2015; Oza et al., 2018) and the Standard Operating Procedure of the Clinical Genome Resource (ClinGen) General Sequence Variant Curation Process, Version 2.0. According to the current consensus, variants designated as “Likely pathogenic” or higher are generally regarded as having potential deleterious effects (Richards et al., 2015; Oza et al., 2018).

### Conservative analysis

The reference protein and DNA sequences for the upstream and downstream regions of the variants in diverse mammalian species were obtained from the National Center for Biotechnology Information (NCBI,https://www.ncbi.nlm.nih.gov/). Multiple sequence alignments were performed using the ClustalW online tool (https://tcoffee.crg.eu/apps/tcoffee/). Conservation of loci across mammalian species was ensured by retaining only conserved regions in alignment.

### Sanger validation

Genomic DNA was extracted from peripheral blood samples and amplified. The amplified DNA was then sequenced using a 3730XL DNA analyzer platform (Thermo Fisher). For validation purposes, samples were collected from the two affected children and their unaffected siblings, as well as from their parents. The primer sequences used in this experiment are listed in Supplementary Table S1.

### The expression data analysis

The gene expression dataset was obtained from the GTEx portal gene tpm dataset (Version 8, https:// www.gtexportal.org/home/). The expression levels of *DOP1A, MBP, PLP1, KIFC2, KIF5B, KIF5C, MON2,* and *MAG* were extracted from the GTEx dataset. The expression patterns were visualized using the R package “ggplot2".

In 2020, Chen et al. investigated transcriptional changes in different brain subregions within a 100-mm diameter around amyloid plaques using spatial transcriptomics in an Alzheimer’s disease (AD) mouse model (Chen et al., 2020). They demonstrated that the co-expression network enriched for myelin and oligodendrocyte genes showed early alterations in spatial transcriptomes. *Dop1a* expression profiles were compared between wild-type and AD mice. Furthermore, the gene expression dataset for human brains during fetal development and after birth was obtained from the Brainspan public datasets (http://www.brainspan.org/). The Pearson correlation coefficient was calculated for each pair of genes, and the expression patterns were visualized using the R package “circlize".

### Protein prediction

The prediction was processed using the online predictor tool PredictProtein (Qiu et al., 2020; Bernhofer et al., 2021), with the reference sequence Q5TA12_HUMAN selected based on WES filtering results. The variant site was compared with the predictions of secondary structure and solvent accessibility. Protein binding sites were predicted using a 3D structure-independent method.

### Protein-protein interaction prediction

The precise conformation of the DOP1A protein in three-dimensional space remains elusive, prompting the utilization of a dependable amino acid sequence-based approach for predicting its protein-protein interactions, as described by Romero-Molina (Romero-Molina et al., 2019).

## Results

### Candidate loci

Three individuals were sequenced, resulting in the mapping of 26415 Mb bases to the target regions for individuals IV-1, IV-2, and III-2, respectively. The mean depth of the target region was 286X while the mean coverage of the target region was 99.8%.

### The Phenotypes of the *DOP1A* affected family

The phenotypes of the affected individuals were collected and resembled the phenotypes of *dop1a* null mutant rats in the research on vacuole formation (VF) rats (Tanaka et al., 2012; 2014). Additionally, Pelizaeus-Merzbacher disease (PMD) patients and PMD-like patients investigated in Alexander Lossos’s research were compared (Lossos et al., 2015). PMD and PMD-like diseases are rare genetic diseases that affect the central nervous system, specifically the myelin sheath and they were reported to be caused by variants in *PLP1/MAG*. Most of the probands and affected relatives were found deficient in movement, linguistic competence, and adaptive behavior (Table 2). The consistent symptoms of PMD manifested in the affected children, such as ataxia, spasticity, nystagmus, and psychomotor retardation. However, the two children exhibited high phenotypical heterogeneity, as observed in PMD patients. The elder brother exhibited abnormal joints and limbs due to severe involuntary muscle spasms, while the younger brother showed milder symptoms. Similar symptoms were also observed in VF rats, although many human tests could not be directly performed on rats.

**TABLE 2:** Symptoms description.

	Ⅳ-1	Ⅳ-2	PMD patients	The VF rats
ataxia	+	+(unbalanced walk before 5 years old, and recovered when grown up)	+	+
spasticity	+ (abnormal joint and limbs, involuntary muscle spasms)	-	+(Spasticity may result in partial paralysis of the arms and legs)	-
Epilepsy	+	+	+	+(Absent-like seizure)
nystagmus	-	+(nystagmus before 5)	+(nystagmus may disappear for some children)	-(no nystagmus but generalized tremor)
psychomotor retardation	+ Dysarthria, no self-care, no self-feeding	+ dysarthria	+(delays in reaching developmental milestones, dysarthria)	No IQ tests (emailing confirmed with the author)
Recovered myelinogenesis	untested	untested	untested	+

IV‐1 The proband child in this family. IV‐2 The younger brother of IV‐1. The VF rats The vacuole formation (VF) rat characterized by generalized tremor, hypomyelination, and periaxonal vacuole formation of the central nervous system identified an homozygous nonsense variant in dop1a on rat chromosome 8.

### Conservative analysis

Multiple sequence alignment analysis of DNA and protein reveals that the loci surrounding the variant are highly conserved across mammal species (Supplementary Figure S2). That indicates these loci are critical for gene function. The amino acid substitution in this region is intolerant and might impact protein function.

## ACMG classification of *DOP1A (NM_001385863.1):c.2561A>G* and evidence of the classification

### The relevance of the phenotype and the gene function

**FIGURE 1:**
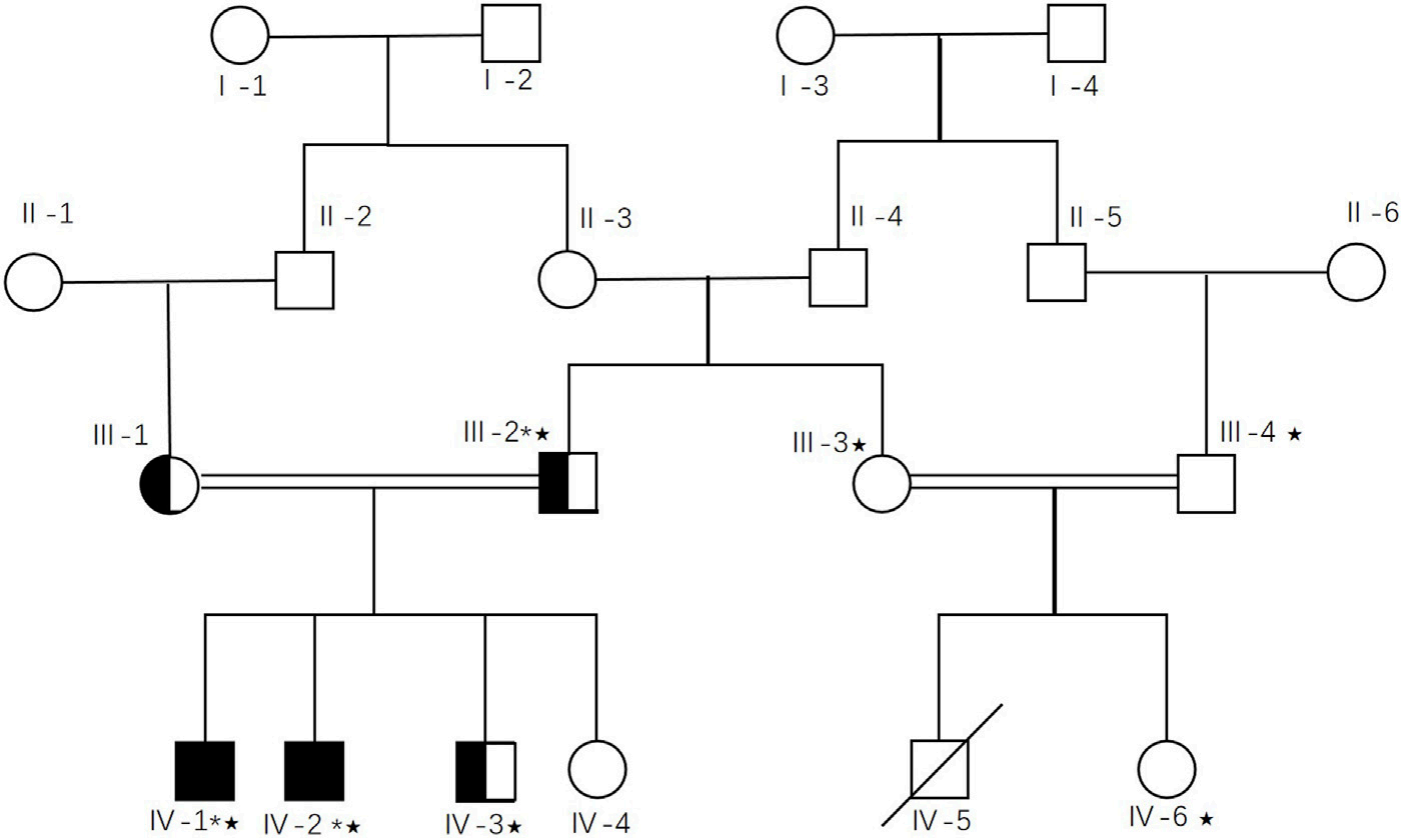
Workflow of candidate gene screening in *DOP1A* affected family.

*DOP1A* has never been associated with any neuronal developmental diseases in humans. However, some studies revealed that the gene could be a potential disease-causation gene of neuronal developmental dysfunctions. As mentioned above, the *dop1a* null mutation rats inbreds were characterized by body tremors, absent-like seizure, abnormal vacuoles in the red nucleus of the midbrain, the reticular formation in the brain stem, and the white matter of the cerebellum and spinal cord, as well as spongy degeneration in the central nervous system which indicates that the gene is a potential target of neuron disorders (Tanaka et al., 2012; 2014). There has been strong evidence that *dop1a* null mutations cause abnormal myelinogenesis, and myelinogenesis is universally considered the primary cause of many neurodevelopmental disorders. Therefore, the gene is relevant to phenotype-associated mechanisms and manifestations. All the evidence for variant classification could be applied to the individual variant-gene-disease interpretation (Figure 1).

### PM2_evidence

According to the gnomeAD exomes database, there was a single allele with no observed homozygotes and no occurrences in the gnomAD genomes database, indicating an extremely low population frequency of 0.00000479. The variant was the most prevalent in South Asia, with a population frequency of 0.0000696. The homozygous variant was determined to provide moderate PM2 evidence for an extremely low frequency of recessive hereditary diseases.

### PM3_evidence

The proband’s father and mother each carry one copy of the variant. For recessive disorders, rare homozygous variants in trans could get no more than 0.5 points according to the ClinGen General Sequence Variant Curation Process Standard Operating Procedure to avoid overclassification. Given the extremely low population frequency of 0.00000479 and the presence of two affected siblings in the family, a PM3_supporting evidence with a score of 0.5 was assigned.

### PP1_evidence

**FIGURE 2:**
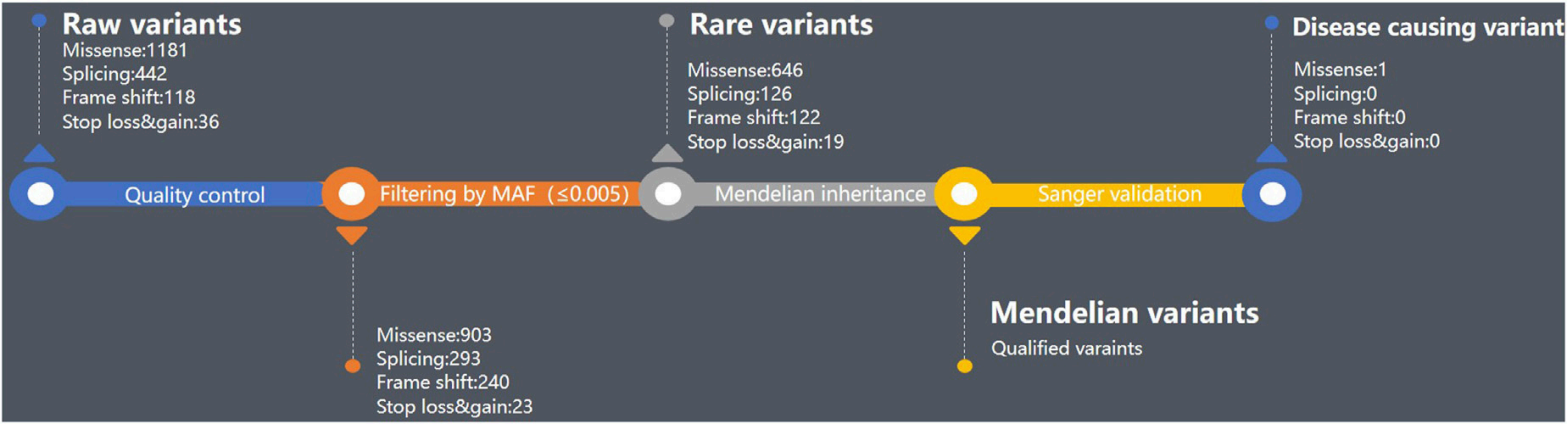
Pedigree tree of the *DOP1A* affected family.

In the co-segregation testing, one affected segregation and one unaffected segregation were identified (parents and the first proband were excluded). Additionally, three relatives in the aunt’s family underwent co-segregation testing, but none carried the variant (Figure 2). Notably, a male cousin died in his infancy with no records of ataxia, spasticity, epilepsy, nystagmus, or psychomotor retardation. None of the *dop1a* null rats showed any symptoms of early death (email confirmed with the author). Therefore, it was inferred that the early death of the male cousin may have been caused by another disease. The LOD ratio was calculated with the following equation for autosomal recessive segregations: $Z\left(Lod\;scores\right)=\log10\left(\frac{1}{0.25^{affected\;segregations}\times 0.75^{unaffected\;segregations}}\right)$ . The Lod score was calculated at 1.102, which provided PP1_supporting evidence (Oza et al., 2018).

### PP2_evidence

There are no pathogenic or likely pathogenic missense variants of *DOP1A* in the Clinvar database, likely because *DOP1A* has not been implicated in any Mendelian inheritance diseases. However, the gnomAD database reveals 919 observed missense variants in *DOP1A*, while the expected number is 1249.5, resulting in a Z score of 3.32. This indicates intolerance to missense changes in *DOP1A*. Thus, the calculated Z score is 3.32. It indicates that *DOP1A* is more likely to be intolerant of missense changes, and missense could be a potential pathogenic mechanism. According to the Clingen criteria (Z score >3.09), these findings support the idea that missense variants in *DOP1A* have a low rate of benign missense variation and that they could be a common mechanism of disease. Additionally, PP2_supporting evidence also supports the variant.

### Classification of* DOP1A (NM_001385863.1):c.2561A>G*

Thus, one PM2_moderate evidence, one PM3_supporting evidence, one PP1_supporting evidence, and one PP2_supporting evidence compose a likely pathogenic variant. The classification supports the high probability that it represents the disease-causing mutation in the affected family.

## Function analysis

### Protein prediction

The variant resulted in a substitution of cysteine for tyrosine at the affected amino acid position, which alters hydrophilicity prediction at this locus. As no crystal or 3D structure of any isoforms of protein DOP1A is available, 3D structure-independent prediction was employed. The prediction indicates the loss of several protein binding sites due to amino acid substitution. The mutant site is moderately conserved and located in a predicted helix region. Although the topological transmembrane structure remains largely unaltered by the variant, the affected locus undergoes a transition from being buried to exposed. Notably, the mutated protein gains an additional N-myristoylation site (GNLRCI) at positions 859–864.

**FIGURE 3:**
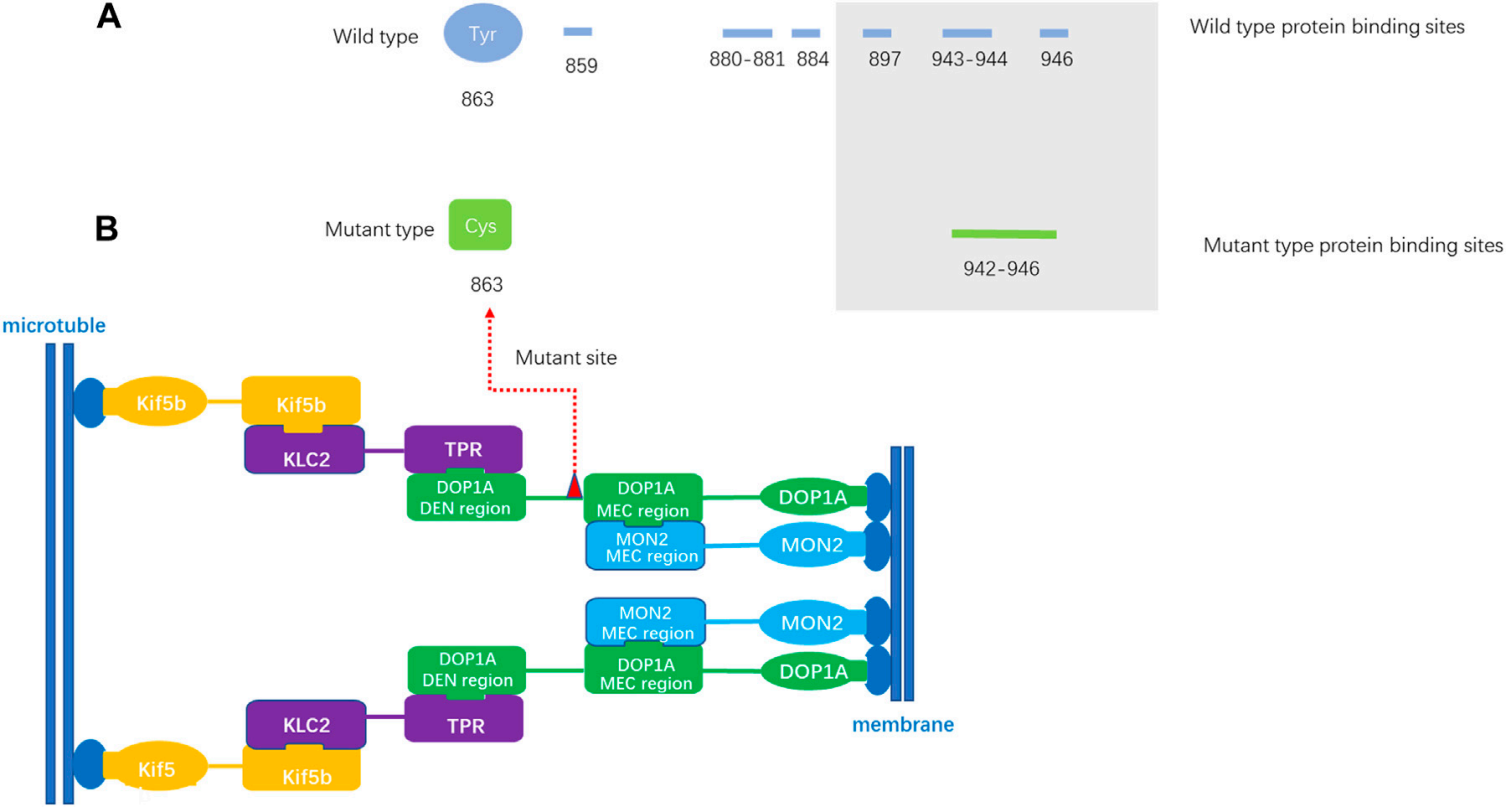
The protein binding site prediction **(A)**. The predicted protein binding sites of wild-type DOP1A. The amino loci 859,880–881,884,897,943–944,946 of wild-type DOP1A are predicted protein binding sites. **(B)**. The predicted protein binding sites of mutant type DOP1A. The amino loci of mutant type DOP1A 942–946 are predicted protein binding sites. MEC region: Mon2 extreme C terminal region (MEC), located in the DOP1A region encompassing residues 894–1247 and co-immunoprecipitants with MON2 C-terminal region of ∼260 residues (1458–1718), which is necessary and sufficient for its interaction. The DOP1A was assembled with MON2 through the MEC interaction region located on DOP1A.The mutation in this family is located near the MEC interaction region and the changes of predicted binding sites might affect the stability of the complex.

In the study conducted by Lei Lu et al., it was observed that the proteins DOP1A and MON2 interact to form a complex that localizes to various cellular organelles, including the Golgi, endolysosome, and endoplasmic reticulum exit site (Mahajan et al., 2019). The N-terminus of DOP1A was found to interact with kinesin-1 (Figure 3). This could result in the disruption of binding sites within the MEC interaction region, further impacting the function of the DOP1A-MON2 complex.

### Protein-protein interaction predictions

**FIGURE 4:**
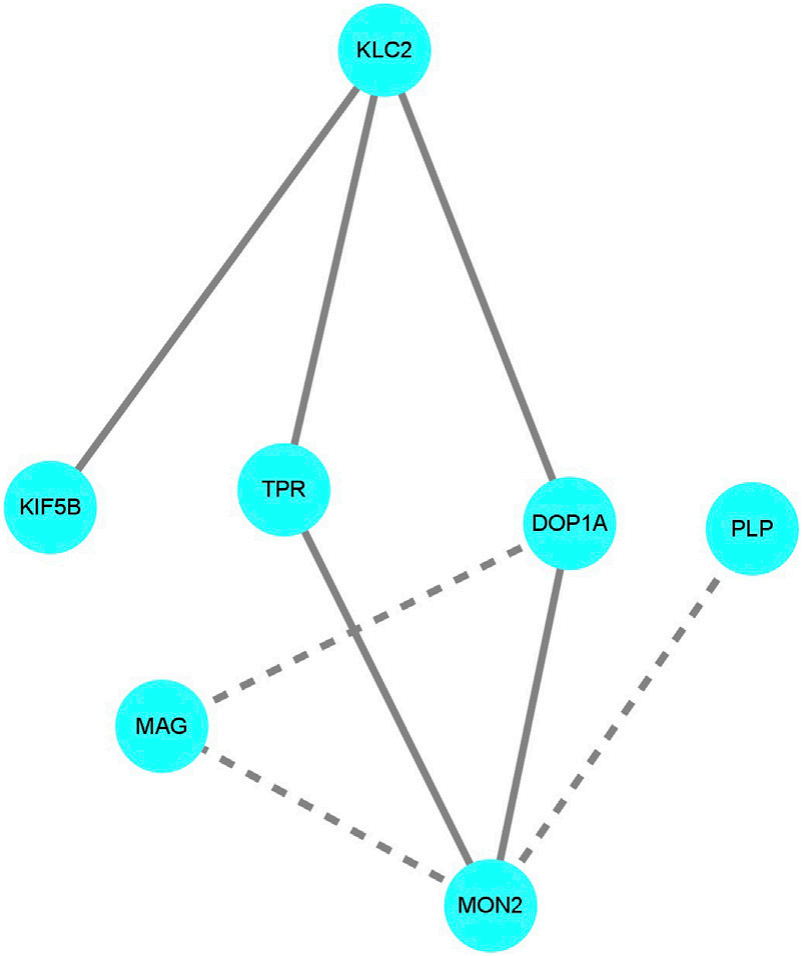
The protein-protein interaction prediction The solid lines indicate the interactions were valid by laboratory experiments in other studies. The dotted lines indicate the interactions were predicted with a probability score larger than 0.5. The DOP1A-MON2 complex was predicted to interact with PLP and MAG.

There are no known protein-protein interactions (PPIs) between PLP/MAG and DOP1A in public PPI databases, and the PPI prediction tool utilized in this study also confirmed the absence of any interactions between PLP/MAG and DOP1A (Figure 4). However, the calculated probabilities of online PPI predictions have identified PPIs between DOP1A-MON2, DOP1A-KLC2, and DOP1A-KIF5B, which were validated by Lei Lu et al.‘s laboratory experiments. The consistency between predictions and laboratory experiments supports the credibility of the method used in our study. In addition, the predicted probability of PPI interaction between DOP1A-MAG was 0.843, whereas the probabilities for MON2-MAG and MON2-PLP interactions were estimated to be 0.536 and 0.887, respectively. These results suggest the high probability that PLP and MAG proteins should be transported by the DOP1A-MON2 complex, and functional variants in DOP1A/MON2 could affect PLP and MAG transportation. The results of the PPI prediction could partially explain the consistency between the phenotypes of the *DOP1A* mutant family and those of PMD/PMD-like patients caused by PLP variants.

### Expression analysis

**FIGURE 5:**
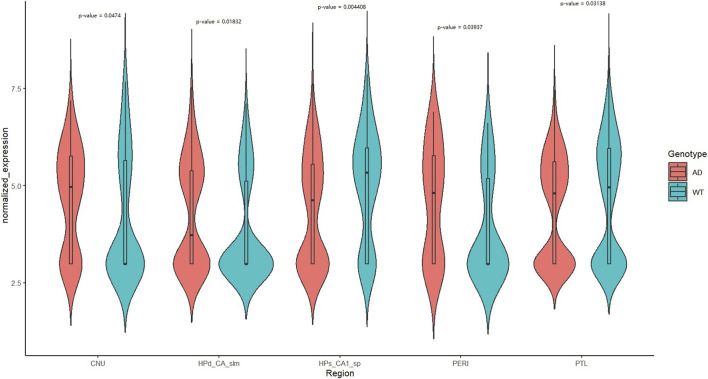
Expression profile of *dop1a* in the wild type and AD mouse brain subregions.

In contrast to the AD mouse model, the normalized *DOP1A* expression levels of wild-type adult mice were relatively lower (Figure 5). The normalized expression levels were compared between brain subregions of wild-type and Alzheimer’s model mice, and five subregions were identified significant differences by the Wilcoxon test. The expression levels were higher in most of the 5 AD mice brain subregions, while they were slightly lower in HPs_CA1_sp and PTL(median_CNU_WT = 2.982974, median_CNU_AD = 4.96435, p _CNU = 0.0474; median_HPd_CA_slm_WT = 2.982974, median_HPd_CA_slm_AD = 3.730511, p _HPd_CA_slm = 0.01832; median_HPs_CA1_SP_WT = 5.331434, median_HPs_CA1_SP_AD = 4.620066, p _HPs_CA1_SP = 0.004408; median_PTL_WT = 4.951096, median_PTL_AD = 4.800058, p _PTL = 0.03138; median_PERI_WT = 2.982974, median_PERI_AD = 4.812775, p _PERI = 0.03937). It has been reported that the myelination in ApoE4 AD patients had been altered by cholesterol_ anomalous oligodendrocytes (Blanchard et al., 2022) and myelin damage was also observed in the AD dementia development process (Dean et al., 2017). The differentiated expression profiles of *dop1a* in AD and wild-type mice indicate the potential correlations between *dop1a* and myelin damage.

**FIGURE 6:**
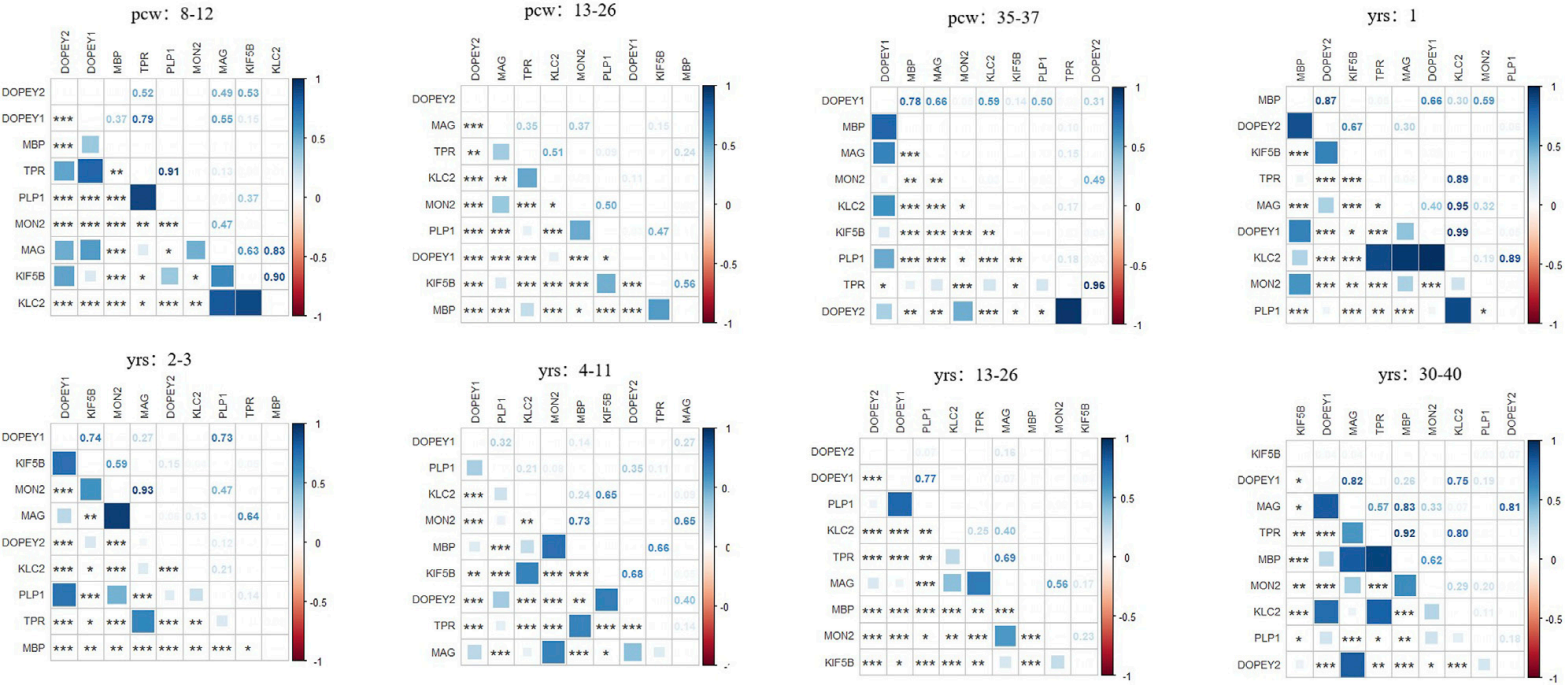
The correlations between DOP1A-MON2 complex and myelin sheath proteins in different developmental stages in the human brain.

To expand upon the relationship between *DOP1A* and myelination, we conducted an analysis of the expression profiles of *DOP1A/MON2* and established myelin protein markers, including *PLP1/MBP/MAG*, utilizing Brainspan datasets (Supplementary Figure S3). Through co-expression analysis of human brain samples across different developmental stages, distinct patterns were identified. During early pregnancy (8–12pcw), the DOP1A-MON2 complex expressed highly, while the three myelin protein markers expressed relatively less. Our results suggested that *PLP1* and *MBP* exhibit mild negative correlations with the *DOP1A-MON2* complex*,* while *MAG* showed no correlation. (r_*DOP1A*_*PLP1* = −0.4,p_*DOP1A*_*PLP1* = 0; r_*MON2*_*PLP1* = −0.47,p_*MON2*_*PLP1* = 0; r_*MON2*_*MBP* = −0.49,p_*MON2*_*MBP* = 0). In late pregnancy, the three proteins positively correlated with *MON2* strongly (r_*MON2*_*PLP1* = 0.56,p_*MON2*_*PLP1* = 0.02; r_*MON2*_*MBP* = 0.64,p_*MON2*_*MBP* = 0; r_*MON2*_*MAG* = 0.67,p_*MON2*_*MAG* = 0). At critical stages, the correlations of PLP1 with the complex disappeared, while the correlations of MBP reached a severe level (2–3_years old: r_*DOP1A*_*MBP* = −0.65,p_*DOP1A*_*MBP* = 0; 13–23_years old r_*DOP1A*_*MBP* = −0.77,p_*DOP1A*_*MBP* = 0) (Figure 6). The co-expressions between *DOP1A* and the myelin protein markers *PLP1/MBP/MAG* throughout the lifecycle, indicated that *DOP1A,* as well as DOP1A-MON2 complex, could play a potential role in the myelinogenesis process. The *DOP1A/MON2/KIF5B/KLC2/TPR* universally expressed in almost every tissue, with the highest expression level in the brain, while the *MBP/PLP1/MAG* is dominantly highly expressed in the brain and nerve but has little expression in other tissues. The two different expression profiles not only indicated the physiological association between the DOP1A-MON2 complex and MBP/PLP1/MAG, but also suggested multiple transport mechanisms in myelin proteins.

## Discussion

*DOP1A* has never been associated with any neuronal developmental diseases in humans. However, some research revealed that the gene could be a potential disease-causing gene for neuron developmental dysfunctions. As mentioned above, the *dop1a* null mutant rats inbreeds were characterized by body tremors, absent-like seizure, abnormal vacuoles in the red nucleus of the midbrain, the reticular formation in the brain stem, and the white matter of the cerebellum and spinal cord, as well as spongy degeneration in the central nervous system, which indicates that the gene was a potential target of neuron disorders (Yamada et al., 1985; Tanaka et al., 2012; 2014) and critical to myelinogenesis which had been universally considered as a cause of many neurodevelopmental disorders (Pacey et al., 2013; Stadelmann et al., 2019).

In Tanaka’s study, the tremor of *dop1a* null mutant rats could recover, accompanied by a concurrent decrease in periaxonal vacuoles. However, hypomyelination existed throughout almost all survival. The expression levels of *PLP1*, *MBP,* and *MAG* were observed to significantly decrease in the white matter of the VF rats compared with the control at 4–10 weeks and slightly decrease in the gray matter. It supported the potential influence of DOP1A on PLP1, MBP, and MAG, which also draws our attention to the similarity of our patients with PMD and PMD-like diseases.

In our study, the potential influence of DOP1A on the myelin sheath proteins was further supported. Protein-protein interactions between PLP/MAG and the DOP1A-MON2 complex were indicated by prediction, and co-expressions with DOP1A-MON2 in critical stages in the mouse brain were identified by the Wilcoxon test. Also, significant differences in *dop1a* expression levels were observed in wild-type mouse and AD mouse brain subregions. The evidence suggested that PLP/MAG could be one of the potential transporting targets of the DOP1A-MON2 complex, and the occurrence of diseases in this *DOP1A* mutant family might partially be attributed to the disruption of normal myelin synthesis caused by aberrant MAG transport.

Our co-expression analysis has revealed the correlations between DOP1A-MON2-kinesin-1 and PLP/MAG at some special developmental stages (from late pregnancy to 11 years old). However, there were weaker correlations between PLP/MAG and the complex after 13 years of age. This suggested that the dysmyelinogenesis due to *DOP1A-*caused PLP/MAG deficiency reached its peak before adolescence and got partial remission after adulthood. The presumption could be corroborated by the research of Mitsuru Kuwamura in *dop1a*-null-mutant rats. They demonstrated that PLP as well as L-MAG and S-MAG significantly decreased from 4 weeks to 10 weeks in the *dop1a-*null-mutant rats but slightly decreased at 20 weeks in the white matter of the spinal cord (Tanaka, 2012). Consistently, the recovery of tremors and the decreasing of periaxonal vacuoles in the *dop1a-*null-mutant rats, as well as the recovery of nystagmus/ataxia in Ⅳ-2, both indicated that there might be other pathways that could complement the dysmyelinogenesis of PLP/MAG partially in adulthood. The diversity of co-expression patterns supports the presumption of the spatiotemporal effect on *DOP1A* of myelinogenesis.

Notably, in our correlation analysis, another DOPEY family gene *DOP1B* showed severe correlations with the three myelin protein markers (8_pregancy_weeks:r_*DOP1B*_*PLP1* = −0.78,p_*DOP1B*_*PLP1* = 0; r_*DOP1B*_*MBP* = −0.51,p_*DOP1B*_*MBP* = 0; 12–26_pregancy_weeks:r_*DOP1B*_*PLP1* = −0.75,p_*DOP1B*_*PLP1* = 0; r_*DOP1B*_*MBP* = −0.63,p_*DOP1B*_*MBP* = 0; 35–37_pregancy_weeks: r_*DOP1B*_*MBP* = −0.68,p_*DOP1B*_*MBP* = 0.04; 2–3_years_old: r_*DOP1B*_*MBP* = −0.66,p_*DOP1B*_*MBP* = 0; 13–23_years_old: r_*DOP1B*_*MBP* = −0.77,p_*DOP1B*_*MBP* = 0; 30–40_years_old: r_*DOP1B*_*MBP* = −0.68,p_*DOP1B*_*MBP* = 0). As described above, the DOPEY family protein *DOP1B* correlates with MBP severely throughout the entire lifecycle, with the highest correlations after 13 years old. It was also observed that there was overexpression of *DOP1B* in the Down syndrome brain regions, and *DOP1B* was considered a candidate gene for Down syndrome (Rachidi et al., 2009). These findings shed light on the potential role of the DOPEY family in the process of myelination during brain development and provide a new potential target for mechanism research and treatment of neurodevelopmental disorders.

Our findings demonstrate that *DOP1A,* as well as the DOP1A-MON2-kinesin-1 complex, could be new candidates for neurodevelopmental disorders due to the potential effect on PLP1 and MAG, which are critical to myelination, but that needs more laboratory-validation of functional and genetic evidence. Due to limitations in local medical resources and social customs, imaging, electrophysiological, and biochemical testing were unable to be conducted on the family members. It prevented us from further describing the physiological characteristics of the disease. The severity of the two affected siblings varies, which is still unexplained in our study. Clinical heterogeneity is also observed in PMD patients with the same variant, suggesting that there might be undiscovered loci that contribute to myelination. The co-expression patterns discussed in our study also support fetal/infantile abnormal myelination caused by *DOP1A*. However, it seems there should be other pathways dominantly regulating myelination by PLP1/MAG expression in adolescence and adulthood, which requires further investigation. The reports that isoforms of PLP1/MAG are expressed differently by developmental stages are consistent with our hypothesis. Additionally, our study revealed DOP1B and MBP co-expression throughout the lifecycle, too. It could be inferred that it is important to conduct more investigations into the DOPEY family for their potential functions in myelination.

## Data Availability

The datasets presented in this study can be found in online repositories. The names of the repository/repositories and accession number(s) can be found below: https://www.ncbi.nlm.nih.gov/, CNSA: https://db.cngb.org/cnsa; accession number CNP0002171).
