# Supplementary material for: Whole exome sequencing identified a homozygous novel variant in DOP1A gene in the Pakistan family with neurodevelopmental disabilities: case report and literature review
Source: Front Genet. 2024 May 16;15:1351710. doi: 10.3389/fgene.2024.1351710 (PMC11137318; doi:10.3389/fgene.2024.1351710)
Supplement: Supplementary file 1 [file Table1.DOCX]

Supplementary

Table 1 the primer sequences in sanger validation

| Primer | Sequence(5’>3’)， |
| --- | --- |
| *DOP1A*-F | TGGACCTGGTGGGACTGACA |
| *DOP1A*-R | ATCTTTCTCCCCTGCTCACA |
