# Supplementary figures and images for: Whole exome sequencing identified a homozygous novel variant in DOP1A gene in the Pakistan family with neurodevelopmental disabilities: case report and literature review
Source: Front Genet. 2024 May 16;15:1351710. doi: 10.3389/fgene.2024.1351710 (PMC11137318; doi:10.3389/fgene.2024.1351710)

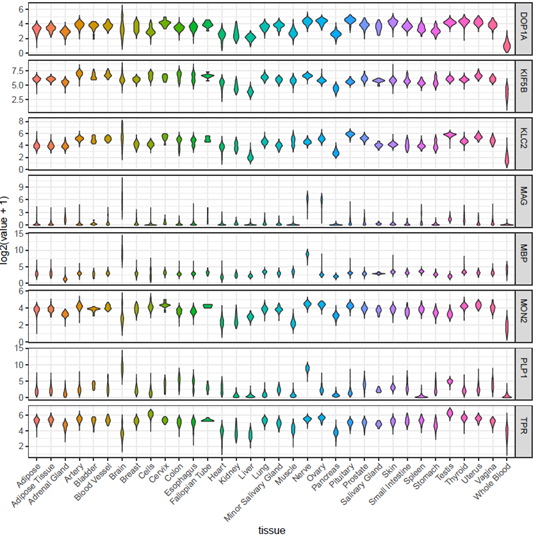

Supplement: Supplementary file 2 [file Image3.TIF]

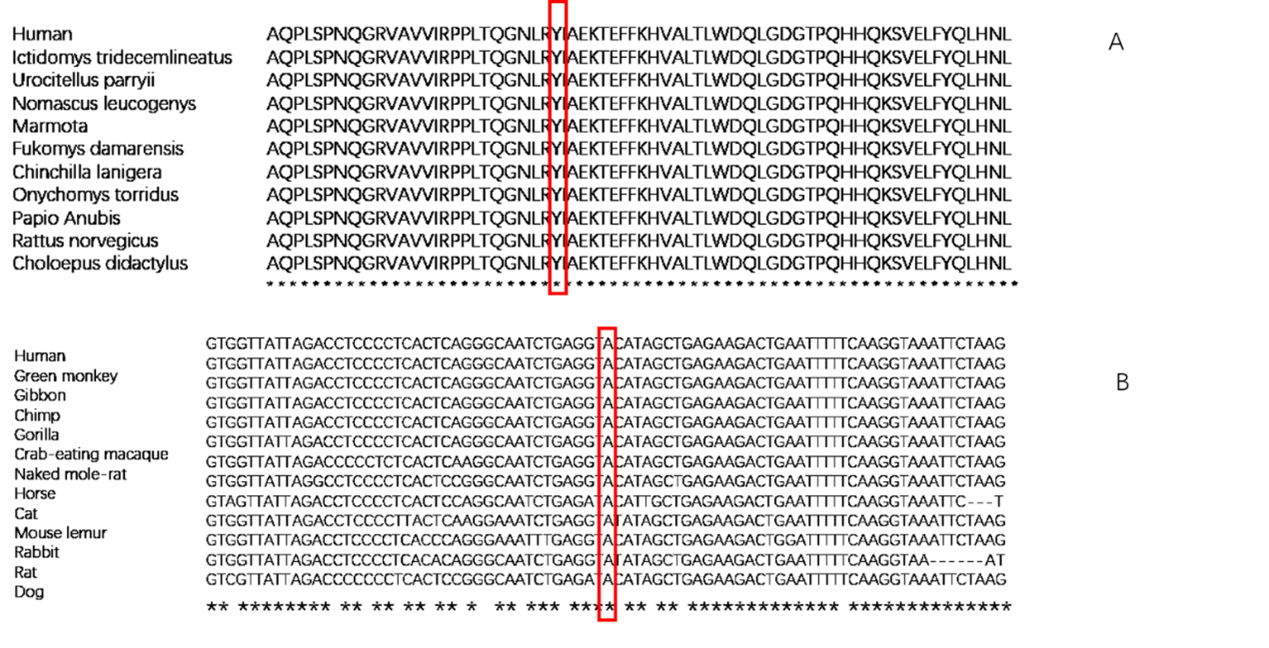

Supplement: Supplementary file 3 [file Image2.TIF]

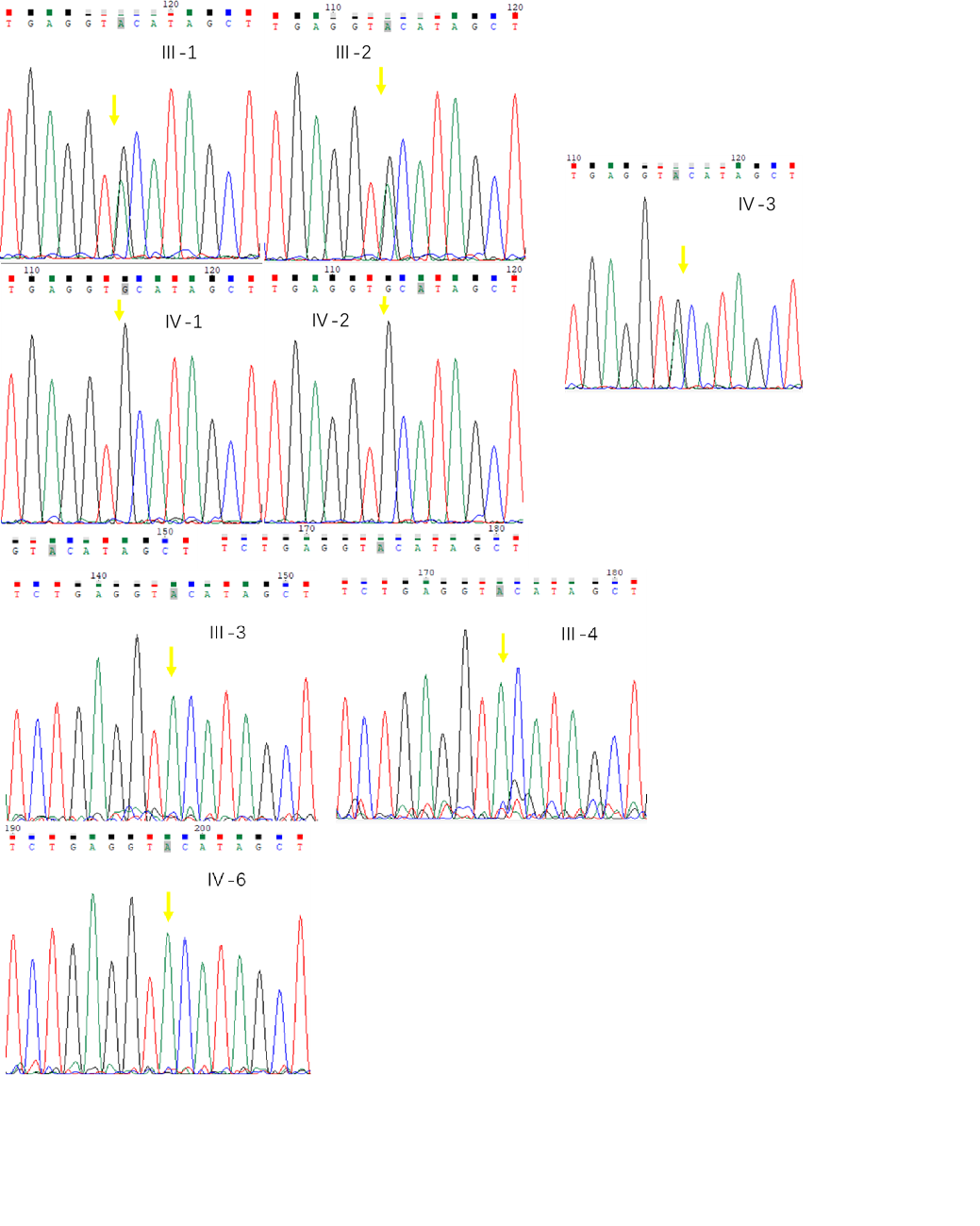

Supplement: Supplementary file 4 [file Image1.TIF]
